# Study protocol and rationale of the “Cogni-action project” a cross-sectional and randomized controlled trial about physical activity, brain health, cognition, and educational achievement in schoolchildren

**DOI:** 10.1186/s12887-019-1639-8

**Published:** 2019-07-26

**Authors:** Patricio Solis-Urra, Jorge Olivares-Arancibia, Ernesto Suarez-Cadenas, Javier Sanchez-Martinez, Fernando Rodríguez-Rodríguez, Francisco B. Ortega, Irene Esteban-Cornejo, Cristina Cadenas-Sanchez, Jose Castro-Piñero, Alejandro Veloz, Steren Chabert, Kabir P. Saradangani, Juan Pablo Zavala-Crichton, Jairo H. Migueles, Jose Mora-Gonzalez, Milton Quiroz-Escobar, Diego Almonte-Espinoza, Alfonso Urzúa, Constantino D. Dragicevic, Aland Astudillo, Eduardo Méndez-Gassibe, Daniel Riquelme-Uribe, Marcela Jarpa Azagra, Carlos Cristi-Montero

**Affiliations:** 1 IRyS Research Group, School of Physical Education, Pontificia Universidad Católica de Valparaiso, Viña del Mar, Chile; 2 Department of Physical and Sports Education, Faculty of Sport Sciences, PROFITH “PROmoting FITness and Health through physical activity” Research Group, Sport and Health University Research Institute (iMUDS), University of Granada, Granada, Spain; 3 Department of Physical Education, Faculty of Sciences of Sport and Physical Education, University of A Coruña, A Coruña, Spain; 4 Physical Education School, Universidad de Las Américas, Viña del Mar, Chile; 5 Faculty of Sport Science, Pablo de Olavide University, Seville, Spain; 6 Departament of Physical Education, Faculty of Education Sciences, University of Cádiz, Puerto real, Spain; 7 Biomedical Engineering Department, Universidad de Valparaíso, Valparaíso, Chile; 8 CINGS, Centro de Investigación en Ingeniería para la Salud, Universidad de Valparaíso, Valparaíso, Chile; 9 School of Kinesiology, Faculty of Health Sciences, Universidad San Sebastián, Santiago, Chile; 10 Escuela de Kinesiología, Facultad de Salud y Odontología, Universidad Diego Portales, Santiago, Chile; 11 Facultad de Educación y Ciencias Sociales, Universidad Andrés Bello, Viña del Mar, Chile; 12 Independent Imagenology Center Quintaimagen, Viña del Mar, Chile; 13 School of Psychology, Universidad Católica del Norte, Antofagasta, Chile; 14 Auditory and Cognition Center, Universidad de Chile, Santiago, Chile; 15 Sports and Exercise Medicine Resident, Universidad Mayor, Santiago, Chile; 16 Universidad Adventista de Chile, Chillan, Chile; 17 Center for Research, Development and Innovation APLICAE, Santiago, Chile; 18 School of Pedagogy, Pontificia Universidad Católica de Valparaiso, Valparaíso, Chile

**Keywords:** Physical activity, Sedentary lifestyle, Fitness, Academic performance, Cognition, Magnetic resonance imaging

## Abstract

**Background:**

Education and health are crucial topics for public policies as both largely determine the future wellbeing of the society. Currently, several studies recognize that physical activity (PA) benefits brain health in children. However, most of these studies have not been carried out in developing countries or lack the transference into the education field. The Cogni-Action Project is divided into two stages, a cross-sectional study and a crossover-randomized trial. The aim of the first part is to establish the associations of PA, sedentarism, and physical fitness with brain structure and function, cognitive performance and academic achievement in Chilean schoolchildren (10–13 years-old). The aim of the second part is to determinate the acute effects of three PA protocols on neuroelectric indices during a working memory and a reading task.

**Methods:**

PA and sedentarism will be self-reported and objectively-assessed with accelerometers in a representative subsample, whilst physical fitness will be evaluated through the ALPHA fitness test battery. Brain structure and function will be assessed by magnetic resonance imaging (MRI) in a randomized subsample. Cognitive performance will be assessed through the NeuroCognitive Performance Test, and academic achievement by school grades. In the second part 32 adolescents (12–13 year-old) will be cross-over randomized to these condition (i) “Moderate-Intensity Continuous Training” (MICT), (ii) “Cooperative High-Intensity Interval Training” (C-HIIT), and (iii) Sedentary condition. Neuroelectric indices will be measures by electroencephalogram (EEG) and eye-tracking, working memory by n-back task and reading comprehension by a reading task.

**Discussion:**

The main strength of this project is that, to our knowledge, this is the first study analysing the potential association of PA, sedentarism, and physical fitness on brain structure and function, cognitive performance, and academic achievement in a developing country, which presents an important sociocultural gap. For this purpose, this project will use advanced technologies in neuroimaging (MRI), electrophysiology (EEG), and eye-tracking, as well as objective and quality measurements of several physical and cognitive health outcomes.

**Trial registration:**

ClinicalTrials.gov identifier: NCT03894241 Date of register: March 28, 2019. Retrospectively Registered.

**Electronic supplementary material:**

The online version of this article (10.1186/s12887-019-1639-8) contains supplementary material, which is available to authorized users.

## Background

Evidence shows a positive influence of physical activity (PA) on brain structure and function, cognition and academic achievement throughout the lifespan [1–5]. At present, international guidelines recommend at least 60 min/day of moderate-to-vigorous physical activity (MVPA) in children older than 5 years, based on its health benefits [5–7]. However, industrialized societies are characterized by high levels of physical inactivity, sedentariness and obesity among children and adolescents [8]. This panorama increases the likelihood of suffering from chronic diseases, dementia and ill-being later in life, facts that are considered of global political concern [9–12].

In this context, schools play a fundamental role because children spend most of their day in these educational establishments. From all of the school subjects, physical education (PE) is the most suited with the potential to increase PA levels of schoolchildren as well as to raise awareness of the need of having a physically active lifestyle. Further, several studies have reported that PE did not impair academic achievement [13]. In fact, increasing both duration and intensity of PE in schools may even enhance both cognition and academic achievement (e.g., mathematics or language skills) [14, 15]. Despite those findings, many schools have decided to maximise the time spent on instrumental school subjects, such as maths or native languages, therefore decreasing PA levels in schools as students pass their course. This strategy could not only be ineffective but also detrimental for academic levels as literature suggests that fitter children hold better brain structure and function [16–19]. Beyond the cognitive improvements, there is no doubt that PA is related to children’s well-being and health [20–22]. Therefore, both health and educational complications could be exacerbated by the limited time of school-based MVPA [23].

In the particular case of Chile, self-reported PA in children has been favourably related to academic achievement [24–26], but studies using objective measures of PA are scarce and inconsistent, even worldwide [4, 27]. Besides PA, both cardiorespiratory and muscular fitness have also been associated with better academic achievement in mathematics and language measured through a national standardized test, the “System for Assessment of Educational Quality” (SIMCE in Spanish) [28]. Likewise, an obese status, excessive screen time, and low nutritional quality have been associated with worse school grades [3, 29–31]. This shows the multifactorial nature of improving cognitive and academic achievement through PA in children.

Mainly physiological mechanism have been used to explain the positive associations of PA level and physical fitness with cognitive function and academic achievement [32]. However, other variables related to educational context, such as the characteristics of PE lessons, the psychological school stress, the sleep quality or the health-related quality of life, have been studied less respect to effect on brain health, and especially in developing countries.

With the aforementioned, it seems necessary and justifiable to carry out a study that tries to cover both health and educational aspects in order to explain the benefits of PA on brain, cognitive and academic performance. This is particularly important in the sociocultural context of Chile due to several key aspects in youth: i) approximately 35% suffer from overweight/obesity [33]; ii) the level of physical inactivity reaches around 70% [23, 34]; iii) there is a high proportion of insufficient academic levels and of students that do not reach minimum learning skills [35], and; iv) high socioeconomic status segregation and market dynamics characterize the national education system [36].

Regarding the last point, the Chilean’s education system is harshly criticized due to the privatization of schools, the voucher system based on average attendance, the creation of incentives and penalties for schools and teachers [37]. These characteristics increase the inequality among students, which initially is originated by a well-known socioeconomic status gap. Indeed, the Chilean educational system estimates the school vulnerability index (SVI), which is an indicator of the degree of educational establishment vulnerability (ranging from 0 to 100, indicating what percentage of students presents in an unfavourable socioeconomic status and therefore, they become priority for the government policies) [38]. Despite the above the Chilean education system is one of the highest-performing in Latin-America but it also presents one of the highest within-country variability in outcomes [39]. In this line, there is a strong relationship between student achievement, socioeconomic status, and financing system of schools [40].

In other context, most of evidence trying to explain the underling mechanisms by which PA improves cognitive and brain functioning have been performed in developed countries [17, 41, 42] and in a well-controlled laboratory settings [43–45]. But, it is also important to develop cross-sectional studies in a more unfavourable sociocultural context as well as experimental studies in a more realistic environment to ensure the integration into the education field.

Experimental trials recently published show that cognitive-related brain activity (e.g., EEG [electroencephalography] oscillations and event-related potentials) is acutely modified after a single bout of PA, boosting cognitive performance in adults [43–46] and children [45]. Whereas there are several mechanisms by which an acute bout of PA can improve brain functioning or cognitive performance, various issues remain unclear [47]. In fact, to date there is not consensus about dose, intensity, duration and type of the PA that better improves brain and cognitive performance, or how long does this effect persists after a single bout of PA [48].

As academic achievement could improve after a single session of PA through a positive effect over working memory and reading [27, 49, 50], it seems to be interesting to study diverse protocols of exercise which can be included within the PE lesson. Despite this idea, with the current quality and quantity of scientific evidence in this field, it is still difficult to visualize a clear direction to establish future recommendations in the school context to improve brain structure and function, cognitive performance and academic achievement through PA.

The purpose of this manuscript is to describe the rationale, design and methods of the “Cogni-Action Project”, which will explore (i) the relationship between PA, sedentarism, physical fitness, brain structure and function, cognitive performance and academic achievement, and (ii) the acute effects of three PA protocols (interval and continuous exercise) and sedentary control condition on neuroelectric activity during resting state and during a working memory and reading task in a large sample of Chilean youth. In addition, this project will investigate the association with other relevant variables related to school context.

## Method and design

### Design and participants

The Cogni-Action Project presents a two-fold design with a cross-sectional investigation and a crossover-randomized trial (ClinicalTrials.gov identifier: NCT03894241). This project has been approved by the Ethics Committee of *Pontificia Universidad Católica de Valparaíso* (BIOEPUCV-H103–2016). In all aspects, this research will be conducted according to the Declaration of Helsinki. Written consent will be obtained prior to participation from the school principal and parents, as well as assent of participants. Any protocol modifications will be communicated and registered on ClinicalTrials.gov.

**Fig. 1:**
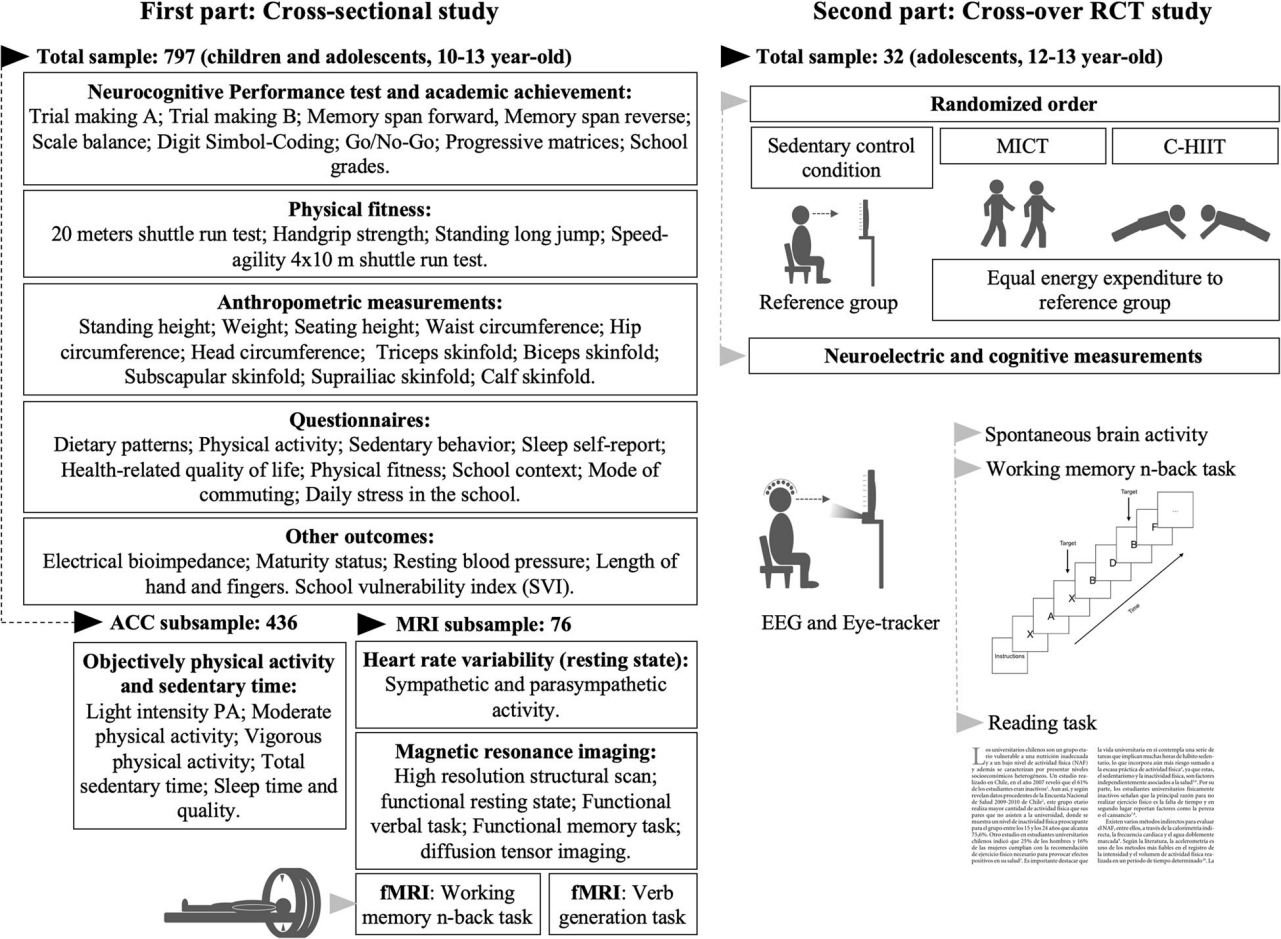
Cross-sectional and cross-over RCT study design

Children and adolescents from 5th to 8th grades (10–13 years old) are recruited from public and private schools in Valparaiso, Chile. This age-group has been selected because of its limited scope, scope, which (tries to) avoid possible methodological bias due to the critical period where preadolescent and adolescent transit through the development of different personal characteristics [51]. It is also an interesting stage of life related to changes in health-related lifestyles, which could have a long-term impact [52], especially in cognition and brain development [53]. A graphical description of the study design, sample and measurements is presented in Fig. 1.

During the first part (i.e., cross-sectional study), measurements will take place in public and private schools, with two visits of 4 h each separated by 8 days. At the first visit, questionnaires, cognitive performance and anthropometric measurements will be assessed, followed by accelerometers placement. During the second visit, physical fitness and other questionnaires will be evaluated, plus accelerometer removal. A detailed description of all evaluations can be found in the measurements section.

Regarding the second part (i.e., crossover-randomized trial), children will be randomized to each protocol session, participating in all of them with two-weeks apart. Thus, they will assist to our gym to perform one of the three protocol and subsequently they will be move to a laboratory where they will undergo EEG and eye-tracking measurements during resting state condition and during two different cognitive tasks. More details can be found in following sections.

### Recruitment and randomization

An open invitation to schools from Valparaiso will be extended after consulting the database of the Chilean Ministry of Education, available at *www.mime.mineduc.cl/mvc/mime/portada*. Then, we will meet with principals to inform about the whole project. After accepting to participate, children, adolescents, and theirs parents will be invited to a new meeting where a full description of the scientific background, objectives, and safety will be given by our research group. During this session, parents and children must sign their participation consent.

### Inclusion/exclusion criteria on total sample

#### Cross-sectional study

##### Total sample

Inclusion/exclusion criteria will be, girls and boys from 5th to 8th grades (10–13 years old) will be included. For ethical reasons, children who present any physical, psychiatric and/or psychological disability will also be included in the cross-sectional study if both children and parents approve their participation. Once the evaluation process is finished, these children could be excluded from the main analysis.

##### Total sample size and power

Power calculation was based on the total enrolment of children and adolescents (5th to 8th grades) indicated by the Chilean Ministry of Education (universe *n* = 951,962) in the year 2016, and assuming an alpha error of 5%, confidence interval of 99, 50% of heterogeneity, and with a 20% dropout. Hence, a total of 797 participants are adequate sample for the cross-sectional part of the project.

##### Subsample

Due to limitation of materials and economic resources, accelerometer recordings and MRI will be assessed in two subsamples. Regarding the accelerometry (ACC) subsample, children will be selected considering the percentage of participation from each school. In the case of the MRI subsample, equal participation of boys and girls will be guaranteed. Children will be included in the randomization if they i) have all previous measurements validated, ii) do not present any visual impairment, so they can correctly perceive the visual stimuli be presented for the functional imaging, iii) have no physical or neurological problem, and iv) are right-handed children, as previous research indicates that brain measures might be different between left- and right-handed people [54]. For the cross-over design, inclusion criteria will be: i) boys aged 12–13 year-old, ii) to have a score > 2 points in Tanner pubertal timing scale [55] iii) normal vision, iv) not being part of the government’s educational integration program (i.e. psychological problems, attention deficit/hyperactivity disorder, depression), v) not having any physical problem that is incompatible with intense PA, vi) not being under psychoactive medications, and vii) to have approved guardians and children’s informed consent.

##### Subsample size and power

From those 797 children, for the calculation of the ACC subsample, we assume an alpha error of 5%, a confidence interval of 99, 50% of heterogeneity, and a 20% dropout, resulting in 436 participants. For the MRI subsample calculation an error of 10% was assumed, with a confidence interval of 90, 50% of heterogeneity, and with a 20% dropout, resulting in 76 participants.

#### Cross-over study

##### Total sample

Inclusion/exclusion criteria will be, boys from 7th to 8th grades (12–13 years old) who not present any physical, psychiatric and/or psychological disability and that they have received the approval of their parents.

##### Total sample size and power

For feasibility reasons, the school chosen for the development of exercise sessions is close to our laboratory. Sample size is estimated according to the mean difference of two independent samples from a randomized controlled trial were a working memory task (2-back) was tested [56]. Children control group achieved a positive variation (∆ = 2.05 ms; SD = 93.1) while exercise group a negative variation (∆ = − 69.45 ms; SD = 91.6) [56]. A loss rate of 20% was considered. Statistical power analysis indicated that at least 32 participants would yield adequate power (i.e., > 80%) and α (i.e., < 0.05), with a detectable variation of 71.50 ms (∆ between experimental conditions – control condition).

### Primary outcomes in cross-sectional study

#### Physical activity and sedentarism

##### Self-report of physical activity and sedentarism

The INTA (in Spanish “Instituto de Nutrición y Tecnología de los Alimentos”) questionnaire will be used to evaluate the usual PA during the week (Monday to Friday) [57]. This scale is composed by 5 items or categories: i) sleeping time, ii) daily time on seated activities or in sedentary behaviours, iii) number of streets walked per day, iv) daily time participating in outdoor recreational activities, and v) time per week participating in exercise or scheduled sports. Each category scores from 0 to 2 points. The INTA questionnaire has shown good convergent validity properties and adequate test-retest reliability in Chilean schoolchildren [57]. Furthermore, Youth Activity Profile-Spain Latin America (YAP-SL) will be used. The YAP-SL is a Latin-American/Spanish adaptation of the 7-day self-administered recall questionnaire. This questionnaire has been translated by the PROFITH group (University of Granada, Spain) [51]. It includes 15 questions about activity at school, activity out of school, and sedentary behaviours.

##### Objective measurement of physical activity and sedentarism

PA and sedentary time will also be recorded through accelerometers, which allow an objective assessment of PA and sedentary time [58]. PA and sedentary time associations with brain and cognitive functions seem to be dependent on the instrument selected to estimate it (i.e., accelerometers vs. self-reports) [27]. Hence, triaxial accelerometers (GT3x, ActiGraph Manufacturing Technology Inc., USA) will be used to determine PA levels, sedentary time, and sleeping time during 24 h (7 days), as it has been recommended in a recent systematic review [58]. The accelerometer kit will be fixed to an elasticized belt and placed on the right side of children’s hip. At least 3 days of the week and 1 day of a weekend will be considered as a minimum recording time for valid registers. A valid day of PA will be considered when the accelerometer is worn for at least 10 waking hours. Accelerometers will be only removed during shower time or swimming activities. Besides, participants should register in a diary-log the time when they remove the device, go to bed, and wake up every day. Thereby, we will examine the time spent on light, moderate, and vigorous PA intensity, as well as sedentary time [59]. The total number of daily steps and steps per minute (i.e., cadence) will also be measured.

In addition, children will be encouraged to wear the accelerometers during the night in order to assess the time and quality of sleep, since it is well accepted that accelerometers provide a convenient way for sleep monitoring [60]. In this sense, the American Sleep Disorders Association supports the use of ACC in assessing several sleep anomalies such as circadian rhythm disorders, insomnia, and limb movements [61]. For all accelerometer data analyses, raw acceleration data will be acquired using ActiLife and then it will be processed using the GGIR analysis package (https://cran.r-project.org/web/packages/GGIR/) for the R programming language.

#### Physical fitness

##### Field-based physical fitness measurements

Physical fitness will be assessed through the ALPHA fitness test battery, which measures cardiorespiratory fitness by 20 m shuttle run test, speed-agility by 4 × 10 shuttle run test, and muscular fitness by handgrip strength, and standing long jump test [62]. To ensure an optimal performance, a brief demonstration of the technique and verbal instructions on how to perform each test will be carried out by researchers.

##### Self-reported physical fitness

Complementary physical fitness assessments will be carried out through the International Fitness Scale **(**IFIS) [63]. This instrument is composed by five items related to the perceived participants’ fitness in comparison with their friends’ physical condition, considering: i) overall fitness, ii) cardiorespiratory fitness, iii) muscular fitness, iv) speed-agility, and v) flexibility. The Spanish language version of IFIS is validated and shows an adequate test-retest reliability [64].

### Brain structure and function: magnetic resonance imaging (MRI)

Brain structural and functional information will be acquired using neuroimaging techniques. All images will be obtained with a 1.5 Tesla MRI scanner (AVANTO, Siemens Medical Systems, Erlangen, Germany). The acquisition protocols are:

- High resolution scanning: This provides structural information of the whole brain. T1-weighted images will be obtained with conventional sequence (MPRAGE, with TE/TR of 2.6/2200 ms), with 1.0 × 1.0 × 1.0 mm3 voxel size. This sequence will allow the study of each subject’s brain anatomy establishing associations between brain volumes and different health outcomes. Total duration of acquisition will be 4 min 32 s.
- Functional magnetic resonance in resting state (fMRI-rs): All fMRI images will be acquired with conventional gradient echo EPI sequence (TE/TR of 50/3000 ms), both fMRI-rs and fMRI-task. Slices will be positioned so that the entire brain is covered. This allows studying the resting state functional connectivity in the brain. Total duration of acquisition will be 6 min 08 s.
- Functional magnetic resonance in task (fMRI-task): Two different fMRI-tasks will be studied: one associated with working memory and another with verb generation. In both cases, the stimuli will be projected on a translucent screen that the child will see using the mirror positioned on top of the head coil that is used to acquire the MR signal. Stimuli will be generated with Python custom scripts, using OpenSesame [65], and words or letters will be presented randomly for each condition accordingly to the programmed design. Each task consists in:
  *Working memory n-back task.* Two different conditions will be performed (0-back and 2-back) [66]. In both cases stimulus will consist in black letters with font size sufficiently big for the child to read easily from within the magnet. For the sake of keeping the task simple, only 8 letters were used: ‘B’, ‘C’, ‘F’, ‘L’, ‘M’, ‘P’, ‘R’, ‘S’, and ‘X’ for the 0-back condition, where the participant will be asked to press an answer button whenever the “X” letter is displayed on the screen. The answer button will be held in the right hand of the participant. In the 2-back condition, subjects will be asked to press the button when the displayed letter is identical to the letter displayed two trials before, where a “trial” corresponds to the presentation of one letter. A total of four blocks (0-back and 2-back) will be performed, beginning with the 0-back condition. Each block initiates with a visual reminder of which task is about to take place: a white “X” letter over a blue background for the “0-back” condition and a black number “2” displayed over a red background for the “2-back” condition. Each block will consist in a sequence of 10 trials for condition; each letter is displayed during 500 ms, with an inter-stimulus interval of 2500 ms (a fixation cross will be shown during each interval). For both control and task conditions, the percentage of target trials is 40%. The total of correct answers will be recorded. The total duration will be 4 min 23 s.
  *Verb generation task.* Two conditions will be implemented: one without stimulus (control condition) and another with stimulus (active task-condition) [67]. In the control condition, a white screen will be presented during 30 s with a fixation cross in the center. While in the active task-condition, ten words will be showed, one at a time, each one presented during 3 s. The participants will be asked to think of a verb, or an action related to each word displayed in the screen. For instance, “bed” will be presented to induce a response such as “to sleep”, “to dream”, or another. All stimuli are presented in Spanish. Five runs of blocks (control–task) will be performed: total acquisition time will be 5 min 08 s.
- Diffusion Tensor Imaging (DTI): This sequence will allow to study the organization of structural (axonal) connectivity and to obtain a quantification sensitive to cytoarchitectonic organization of white and gray matter. 30 diffusion-weighting directions will be used, and with b values of 1000 s/mm^2^ the whole brain will be covered. Total procedure duration will be 4 min 02 s.

### Cognitive performance

#### NeuroCognitive performance test (NCPT)

**Fig. 2:**
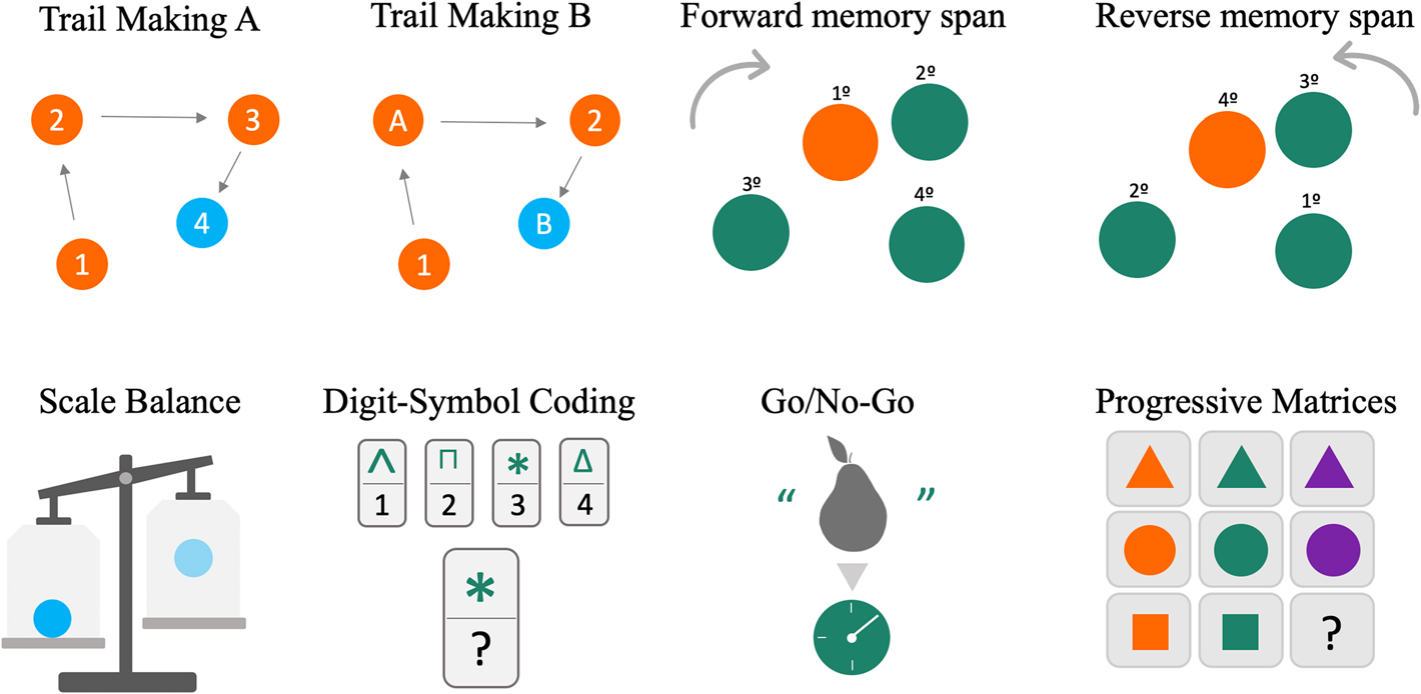
Eight cognitive tasks that conform the neurocognitive performance test

The NCPT (Lumos Labs, Inc.) is used to assess cognitive performance [68]. It is a brief, repeatable, web-based platform of cognitive tasks intended to measure functioning across several cognitive domains including: working memory, visuospatial memory, psychomotor speed, fluid and logical reasoning, response inhibition, numerical calculation, and selective and divided attention. The NCPT has demonstrated adequate reliability and validity as a measure of cognitive performance, and in good concordance with pencil-paper assessments [68]. Eight cognitive tasks will be assessed in this study (Fig. 2): i) “Trail Making A” and ii) “Trail Making B” estimate attention, cognitive flexibility and processing speed, and are based on the Army Individual Test Battery [69] and the Halstead-Reitan Battery, respectively [70]; iii) “Forward Memory Span” and iv) “Reverse Memory Span” determine visual short-term and working memory, respectively, and are based on the Corsi Blocks tasks [71]; v) “Balance” based on Wechsler Intelligence for Children IV [72] and Piagetian Balance Beam Tasks [49], which judges for quantitative and analogical reasoning; vi) “Digit Symbol Coding”, based on the Digit Symbol Substitution Task [73] and evaluates processing speed; vii) “Go/No-Go” task that checks for response inhibition control and processing speed; and finally viii) “Progressive Matrices”, based on established matrix reasoning assessments [74] and is designed to assess problem solving and fluid reasoning.

### Academic achievement

#### School grades

Final school grades will be obtained from the official records. The grades of mathematics, language, natural sciences, social sciences, English, PE, technological sciences, art and music will be consulted. Additionally, the grade point average (GPA) will be calculated.

### Secondary outcomes

#### Anthropometry

Initially, it will be performed with basic measures of weight (OMROM, HN-289-LA, Kyoto, Japan), height and seated height (SECA, model 213, GmbH, Germany); besides, waist, hip, and head circumference; and skinfold thickness of triceps, biceps, subscapular, suprailiac, and calf will be measured. Head, waist and hip circumference will be measured with an inextensible tape (Lufkin, Apex, NC). Waist circumference will be taken in a horizontal plane, at the level of the natural (minimal) waist and taken at the end of a normal expiration. Hip circumference will be measured at the maximum protruding part of buttocks at the level of the greater trochanter with children wearing minimal clothing, standing with their feet together.

#### Body composition

Electrical bioimpedance will be used to measure body composition, employing an Inbody S10 device (Biospace, Seoul, Korea). The participant will be seated, with eight electrodes placed in the body, two in each hand, and two in each ankle. Each participant will lay seated for a 5 min rest period before the procedure.

#### Maturity

The maturity status will be estimated through two methods. First, peak height velocity (PHV) will be calculated through Moore and colleagues’ equation [75]. Complementarily, Tanner staging will be used to assess the degree of pubertal development. The participant will select his/her state on a scale from 1 to 5 maturity degrees.

#### Digit ratio

Recently, length of fingers, and specifically the ratio of the second and fourth digits of the hand (2D,4D) has been strongly related to prenatal testosterone exposure [76]. We included this measurement due to the relationship of testosterone with brain development and the association of 2D:4D with brain volumes and intelligence quotient [77, 78]. Therefore, this novel and simple measure will be calculated according to criteria of digit ratio measurement guide [79]. Finally, analyses will be made with Autometric software whose reliability has been previously demonstrated [80].

#### Blood pressure

Resting blood pressure will be assessed after 5 min of rest by an automated device (Omron HEM-7130®). Two readings will be taken with a difference of 3 min between each one. With the participant seated, left arm will be placed on the table and the bracelet will be fitted around the arm, above the elbow, and aligned with brachial artery. A pediatric bracelet will be used when necessary.

#### Heart rate variability

Prior to MRI, an objective estimation of autonomic balance will be measured through the heart rate variability (HRV). Participants will be placed in a quiet room without noise or any other distracting stimuli (e.g., light) and HRV will be measured completely at rest in a supine position. A minimum of 12 min of recording is needed. A practical finger device, SENSECORE, which has shown good reliability and accuracy in children will be used [81]. Analysis will be made using the Kubios HRV software (University of Eastern Finland, Finland) [82].

#### Sleep self-report (SSR)

Sleep patterns will be assessed using the **sleep self-report** [83]. This questionnaire is composed by 26 items and four subscales: 1) Sleep quality; 2) Sleep anxiety; 3) Bedtime refusal; and 4) Sleep routines. The Spanish version showed adequate psychometric properties and good internal consistency (w = 0.85) [84].

#### Mode of commuting

Commuting to and from school will be evaluated by self-report. Participants will answer the following questions “How do you usually travel to (from the) school?”, “How many distance there is between home and school?” and “How much time does it take to get to (from the) your school?”. The responses to the first question can be: by walk, bike, motorbike, car, bus, several transports or other transports (requesting it in those cases). These questions come from the previously validated “PACO” (in Spanish “Pedalea y Anda al Colegio”) questionnaire [85, 86].

#### School context physical activity

The school PA questionnaire (SPAQ) is a Chilean validated questionnaire referred to different aspects of school environment and PE lessons [87]. Questions refer to sport practices, possibilities of outdoor environment, body expression development, PA promotion, development of self-care, development of active life, didactic of lessons and any kind of other school activities.

#### Dietary patterns

In order to estimate quality of nutritional intake and nutritional habits two food frequency questionnaires will be used: the Mediterranean Diet Quality Index (KIDMED) for children [88] and the Healthy Eating Index (HEI) according to national guidelines [89]. Also, questions about quality of breakfast will be performed. The KIDMED index has been previously validated and it is widely used in childhood [88].

#### Health-related quality of life

Kidscreen-27 questionnaire will be used to measure health-related quality of life [90]. This questionnaire was designed specifically for young people aged 8–18 years and consists of 27 items and five dimensions: i) physical well-being, ii) psychological well-being, iii) its relationship with parents and children autonomy, iv) social support and peers, and v) school environment. The Chilean version of Kidscreen-27 has shown an adequate internal consistency (*α* = 0.89) and high reliability [91].

#### Daily stress in the school context

A scale of stress will be used to assess the daily stress within the school context. This instrument consists of 18 items, arranged in 3 dimensions: i) stress of relational violence, ii) academic stress, and iii) environmental stress. The validation of this scale has shown good internal consistency (*α* = 0.90) and adequate validity properties in Chilean children and adolescents [81].

#### School characteristics

Several characteristics of school will be registered such as SVI, scholar schedule, public or private administration, location, and educative project.

#### Parental reports

Also, through self-report from parents we will obtain different variables such as family socioeconomic status, nutritional status, parents’ educational level, height and weight of participant at birth, possible complications during pregnancy or at birth and other sociodemographic information. Additionally, parents will fill the Children’s Body Image Scale (CBIS) for preadolescent and children according to their own perceptions about their children.

### Design of cross-over acute trial

The design of the second part of the project will consist of a cross-over trial where the participants will perform, on different days, three different PA protocols with equal energy expenditure fashion in a random order. Before starting this part, schoolchildren will be evaluated by a physician (preparticipation physical evaluation), under previous authorization of their parents. The examination will consist in the realization of a complete clinical history and a general physical examination, with the purpose of verifying their health status and avoiding possible injuries or illnesses during PA protocols. Any adverse events occurring during the trial will be documented and submitted to the physician, which will analyze the exclusion of the participant from the trial.

Each child will visit our laboratory three times, every 2 weeks to ensure a “wash-out” period with the purpose of mitigating the PA effect from each protocol. The order will be determined using a computer-generated ordering system. The random sequence was generated by the web-based Research Randomizer (randomizer.org). A staff member responsible for recruitment will perform randomization. Participants will assist to the three different sessions at the same weekday (e.g., Monday) and at the same daytime to avoid differences in preceding school activities or circadian rhythms [82]. Participants will undergo a “Sedentary condition” (SC), sitting and watching a documentary on TV as has been used previously [82], and two different PA protocols consisting in “Moderate-Intensity Continuous Training” (MICT), continuous outdoor brisk walking/running; and “Cooperative High-Intensity Interval Training” (C-HIIT), composed of a circuit training with a partner (physical education teacher) (Fig. 1). These three protocols have been chosen in view of what corresponds to types of activities usually performed in schools. Thus, SC mimics the time spent during any academic lesson (i.e. mathematics or language lessons); MICT represents the typical activity in a PE class; and C-HIIT has been selected since it is postulated as an exercise protocol that could possibly be implemented in thethe PE class [92]. The three protocols are designed so that energy expenditure is equivalent between them, to eliminate differences in the energy expenditure as a possible confounding factor. Thus, the duration will be different among protocols.

#### Equal energy expenditure fashion and conditions

Energy expenditure by activity will be estimated through metabolic equivalents (METs) which is defined as the amount of oxygen consumed while sitting at rest, and it represents a practical way of expressing the energy cost of physical activities as a multiple of the basal metabolic rate (BMR) [93]. As estimation of METs strongly depend on body weight the following equation will be applied with the intention to personalize the total energy cost to each participant: Total Energy Cost (kcal) = METs x BMR (kcal/min) x duration (min), where the BMR for boys between 10 and 18 years is predicted using the equations: BMR (kcal/min) = [17.686 x weight (kg) + 658.2]/1440 [94].

According to the last “Youth Compendium of Physical Activities” [95] children above 10 years spend 1.3 METs sitting “watching TV” (similar to sitting in school) as Sedentary condition (SC), while that in a “walk self-paced brisk” as the Moderate-Intensity Continuous Training (MICT) protocol they will spend 5.0 METs, and for a vigorous PA such as “obstacle/locomotor course–vigorous” exercise as a Cooperative High-Intensity Interval Training (C-HIIT) they will spend 8.6 METs. In this sense, SC will be the referential group, and then both MICT and C-HIIT will have the same energy cost that SC.

Finally, to confirm children’s physical intensity each one will use a heart rate monitor (Polar H10) to encourage maintenance of the appropriate exercise intensity, which will be connected to an iPad or mobile application (Polar Team).

#### Characteristics of each PA protocol

##### SC

Each adolescent will be seat in a room within the laboratory with a TV in front of. They will watch a documentary about nature during around 90 min because in Chile any traditional academic subject as math or language last this time. Temperature, light, and sound of the room will be controlled.

##### MICT

Each adolescent, together with a personal trainer of our team will go for a walk self-paced brisk. This PA will be performed outdoor because is more realistic to school activities. The MICT protocol must be done to moderate intensity, corresponding to 60% of heart rate maximum [82].

##### C-HIIT

Each adolescent together a personal trainer will do a circuit training based on a collaborative fashion. The specific protocol has been published previously [92]; in brief, it consists in four series of four cooperative exercises which combining cardiorespiratory, speed–agility, and coordinative training exercises because these are the fitness components that mostly enhance cognitive capacity in adolescents [56, 92, 96]. The session will be organized with work-to-rest ratios of 20:40 s. Both, MICT and C-HIIT protocols include a short 4 min warm-up (running, sideways movements and dynamic stretching at medium intensity) and 4 min of cool-down. The C-HIIT protocol must be done to high intensity, corresponding to ≥85% of maximum heart rate [97].

After each PA protocol, the participants will undergo EEG and eye-tracking measurements as follows: 1st, resting state spontaneous brain activity will be assessed during 3 min with EEG only, and subsequently, eye-tracking will be registered in parallel with EEG while participants perform the cognitive tasks (a working memory task and a reading task). The measurements will start between 20 to 25 min after the end of each condition. In this way, we will emulate a school recess in the Chilean context, before the start of the next subject. All measures will be carried out in the “Laboratorio Lenguaje & Cognición ELV”, which belongs to the Literature and Language Sciences Institute of the Pontificia Universidad Católica de Valparaíso. To avoid subjective influence of evaluator, when the participants go to the laboratory, the evaluator will be blinded according to which PA protocol participants performed.

Regarding optimize participant retention, parents or legal guardian will be contacted and reminder by phone calls, or email when the participant loses any of the three scheduled sessions. Then, missed sessions will be reprogramed according to availability of participants and respecting study design. An inventory of each section of this trial is available in the SPIRIT checklist (Additional file 1).

### Neuroelectric and eye-tracker measurements

#### EEG measurement

A B-Alert X24 device for EEG (Advanced Brain Monitoring, California, United States) will be used, which consists of 24 active electrodes that minimize the noise of electrical devices outside the biological processes of interest, ensuring good signal quality. Two channels will be used to register electrooculographic activity, to better discriminate between true electroencephalographic and electrooculographic activity associated with eye movement and blinking. Recordings will be done at a sampling rate of 256 values per second, with a band-pass filter between 0.1–100 Hz, and a notch filter of 50 Hz to eliminate the noise of the surrounding alternating current in the room. It should be noted that this equipment is wireless, which makes it more convenient and faster to mount on participants.

#### Eye-tracker

A Tobii Pro TX300 (Tobii, Stockholm, Sweden) will be used to track eye movements directly through a light sensitive camera near the infrared spectrum. This equipment studies the visuomotor characteristics during the process of reading and the trajectories of the look around each word. In addition, it will serve to define the exact moments at which these words are read and thus be able to synchronize the EEG record to calculate an average signal (N400) that reflects the brain’s processing of language. The same device records raw values of pupil size (diameter), saving all data with a sampling rate of 300 Hz.

Specific software of each company will be used for the registration of the EEG and eye-tracker signals, synchronized through digital pulses sent via a parallel port, from the PC housing the stimuli presentation software. This will allow having a single timeline for both registers. The data will be analyzed through analysis packages and custom scripts written in Matlab. For the spectral analysis of EEG during the recording at rest condition, the EEG signals will be filtered between 7 and 13 Hz, and the Hilbert transform method will be applied, which allows calculating accurately and without restrictions of precision both the dominant frequency and the oscillatory amplitude of a frequency band. This method has not been applied before in this line of research, and has several benefits with respect to conventional spectral methods such as the Fourier method. Then, the results of each channel will be averaged to have a more robust overall measurement of the peak of the alpha wave, and its amplitude changes when opening / closing the eyes. For the cognitive tasks (working memory and reading), the amplitude and latency of the corresponding event related potentials will be quantified (P300 and N400, respectively).

### Cognitive tasks during neuroelectric and eye-tracker measurements

#### Working memory task

A N-back task will be run through E-Prime 2.0 software synchronized with the electroencephalographic recording system and the eye-tracker, to measure neuroelectric activity and pupil size diameter time-locked to the presentation of each stimulus (targets and non-targets). The protocol is very similar to the one previously explained for the 0-back and 2-back conditions during fMRI assessment. The main difference of the present task with respect to the fMRI’s N-back task is that children must press in the keyboard the number “1” when they detect a target stimulus or the number “2” for non-targets. This type of task generates an EEG potential related to cognitive processing, characterized by a positive deflection that peaks roughly around 300 ms (P300), which has been widely studied in various types of tasks that require conscious attention, in any perceptual modality [98, 99]. In other words, it is a potential related to events that are consciously perceived, which does not depend on the stimulated perceptual path, but rather on the need to reallocate cognitive resources and develop appropriate responses according to the ever-changing context. To isolate the EEG patterns of P300 in working memory, we will subtract to the result of a 2-back task, the one of a 0-back. The P300 event-related potential is expected to emerge especially in the average EEG signal time-locked to the onset of detected targets stimuli [100, 101]. Artifacts such as electrooculographic signals will be subtracted from the data with an algorithm implemented in EEGLAB [102], an EEG analysis toolbox running in MATLAB (Mathworks®). Manual rejection of single trials will be considered based on visual inspection of artifacts in data.

#### Reading task

Language processing will be assessed by a reading comprehension task where short paragraphs will be presented to be read normally. The eye-tracking technique will be employed to record eye position, movement and pupil diameter, simultaneous to the EEG record. Participants will seat in front of a monitor, at approximately 60 cm, placing their chin on an ergonomic stand piece, which helps to decrease head movements and improves eye-tracker signal precision. The reading task will be presented with the latest version of Tobii Pro Lab. After calibrating the system, each child is given a brief contextualization of the topic, before they start to read, knowing that later they will be asked seven comprehension questions with varying degrees of complexity. The participants must indicate the correct answer among four alternatives. It should be noted that three literary texts will be used, presented in a random fashion (1 per exercise condition). These texts will have the same difficulty, the same distribution of areas of interest, similar amount of words, and will be previously validated by a panel of experts. The reading of each word is expected to generate an EEG negative potential with latency close to 400 ms (N400). The amplitude, topographic distribution and latency of this potential are electrophysiological neuromarkers of linguistic ability in general, and the ability to construct meaning from words [103]. The eye-tracker will be used to study the ocular trajectory during reading and establish the moments when words are read. EEG and eye-tracking will be registered simultaneously to record eye movements related to reading, giving measures such as reading speed, fixation durations, number of fixations, among others), and to measure eye fixation related EEG potentials associated with cognitive load while reading words [50]. The reader should note that this potential coincides experimentally with the N400, so they may represent different brain processes superimposed, or be essentially the reflection of the same mechanism.

### Primary outcomes in cross-over study

#### Spontaneous brain activity in resting state

After the exercise protocol and before the cognitive tasks, spontaneous brain activity will be measured with EEG while children rest in a seated position, in order to measure the peak frequency [43] and reactivity of the alpha wave [104, 105]. The dynamics of the alpha peak frequency (between 7 and 13 Hz) during a 3 min period will be calculated by means of a Hilbert transform [106]. Then, an average value and trend of the alpha peak frequency will be calculated. It has been postulated that the higher the alpha peak frequency, the greater general cognitive capacity and processing speed are [44]. We focus on alpha waves because this brainwave has traditionally associated with different theories of attention and conscious perception [107, 108]. The frequency of the alpha wave has been discovered to increase after a session of cognitive tasks [109–111]. Additionally, the power and frequency of alpha brainwaves prior to cognitive evaluation have been correlated to performance [112]. If PA exerts a positive effect over cognitive performance, a single session of PA should also cause an increase in alpha peak frequency. In fact, this was found by Gutmann et al. in two consecutive studies [43, 44]. However, a statistical relation between PA-induced increments in alpha peak frequency and cognitive performance is missing.

#### Working memory performance

Hits, misses, omissions, and accuracy will be calculated for targets and non-targets of the n-back task, as well as response latency.

#### Reading task

Seven questions with varying degree of comprehension complexity will be given after the texts are read. For every question there will be four alternatives to choose as the correct answer.

#### Cognitive load

We group several measures on this category because all of them have been related with the concept of cognitive load. The first two come from the EEG, and the other two from the eye-tracker.

##### P300

The P300 event-related potential associated with the different type of stimulus in the n-back (target and non-target) will be quantified according to amplitude and latency of the peak.

##### N400

This linguistics-related event-related potential also will be described according to latency and amplitude. Because it will be synchronized with the eye fixation on words during the reading task, it is conceptually a type of fixation-related potential.

##### Rapid pupil size variations

Pupil reacts to cognitive requirements and it has been associated with increases in cognitive load during various tasks including working memory and reading [113, 114]. The amount of high frequency variations in pupil diameter is estimated by a novel Index of Cognitive Activity [114], which increases with cognitive load. This measure can be represented as a continuous variable in time thanks to the high sampling rate of the eye-tracker. The Index of Cognitive Activity will be measured during the n-back and reading tasks.

##### Word fixation times

The length of visual fixations also has been associated with the processing time that the brain dedicates to elaborate a cognitive interpretation of perception. Average and standard deviation of fixations around words will be calculated for the reading task.

### Data analysis plan

#### Cross-sectional study

Descriptive data from participants will be summarized separately for boys and girls, and presented as means and standard deviations for continuous variables and as percentages for categorical variables. T-test or chi-square analysis will be used to establish sex differences where appropriate. Also, interaction analysis by sex will be performed previous to the main analysis.

Given that the first aim of the project is to associate PA, sedentarism, and physical fitness with brain structure and function, cognitive performance and academic achievement; general linear models will be employed. Additionally, multilevel random-effects models will be used. Furthermore, as knowledge about this relationship in Chilean population is scarce, mediation analyses will be performed with other relevant variables related to school context. The significance threshold for all analyses will be *p* < 0.05.

#### Cross-over study

Differences among condition will be calculated using general lineal models (95% confidence) and estimating the Hedges effect size [115]. Strength of effect will be assessed according to the following interpretation: trivial (0–0.19), small (0.20–0.49), medium (0.50–0.79) and large (0.80 and greater) [116]. The significance threshold analyses will be *p* < 0.05.Regarding missing data, it is anticipated that missing data could be due to faults in the EEG and eye-tracker, and thus will be missing at random. For this reason, multiple imputation will be used to address this issue.

In addition to performance in the task of working memory and reading, several neurocognitive variables (amplitude and latency of both potentials, frequency of the alpha wave, and variation of its amplitude with the opening of the eyes) will be studied statistically using a model of mixed effects, which initially will use the factors: subject, exercise protocol and cognitive ability (high or low performer). Subsequently, it will be extended to other factors (the rest of the measures of the Cogni-Action Project, such as body composition, sexual development status, anthropometric measures, socioeconomic, brain structure, etc.), to establish which factors and interaction between them have greater statistical significance in the acute effects of PA on cognitive and academic performance.

##### Data management

Data for all participants will be stored in coded form. The codification will be perform by a researcher of team and will be stored online to avoid any issue. The senior researcher will be control the shared of data with another researchers. As the intervention have only acute characteristics, any adverse event will be controlled immediately by researcher team in the place of is carry out the intervention.

##### Dissemination of results

The research team will be access to both cross-sectional and RCT dataset. Additionally, the senior researcher (CC-M) could share the database previous request. Each participant will know your general results if parents ask it and any medical findings are timely informed. Outcomes from the study will be published in peer-reviewed academic journals as well on scientific meetings and social media.

## Discussion

### Expected results and transfer to the school context

Nowadays, both education and health are two of the most relevant concerns worldwide [117]. Thus, during the last years, a great interest has generated to establish the association among PA, sedentarism, and physical fitness on brain structure and function [4, 53], assuming that to some extent; it could be transferred to the scholar success. However, there is a lack of scientific evidence based on advanced neuroimaging and neuroelectric techniques (e.g., MRI, EEG), and objective methods to measure PA (e.g., ACC) in developing countries.

Regarding the educational context, the contributions of this project will cover two important aspects in order to improve both academic achievement and cognitive performance. The first aspect is related to establish general recommendations due to the poorly understanding about PA dosage linked to brain health [118]. For this, a complete analysis will be performed with the intention to understand which factors, such as time and type of PE lessons and recesses, intensity of PA, effectiveness of PE, time on sedentary behaviours, physical fitness components (e.g., strength, cardiorespiratory fitness or speed-agility fitness), time of day to perform PE (e.g., morning or afternoon) are more related to a better academic achievement and cognitive performance. Furthermore, we will test the mediation influence of vulnerability index, type of schools (financing system), perception of school stress, among others. This is an important topic to study due to the great social inequalities in Chile.

The second aspect, it about the negative acute physiological effect of sedentary behaviours (typical class of school), in contraposition with the hypothesised positive influences of different models of PE lessons on working memory and reading [48]. Thus, PE could be used as a learning enhancer for important subject such as mathematics, languages, and others [27]. Hence, this project will also allow advancing in the understanding about the underlying bases of acute effects of a single bout of PA on spontaneous brain activity and cognitive performance during different cognitive task. Likewise, whereas our proposed intervention is not in a real school environment, the design of this experimental study has based its methodology emulating the times and activities that are usually carried out in schools.

Finally, we hope that the results of this project will help to generate future programs and interventions both at the ministerial and at the local level (schools) in order to improve the physical health and cognitive and academic performance of schoolchildren.

The “Cogni-Action Project” will try to identify efficient ways to improve academic achievement and cognitive performance in youth trough PA during the school day as well as during the rest of the day. To our knowledge, this project will be the first cross-sectional study using neuroimaging and objective PA measures in Latin-America, which also incorporates another study about the acute effects of PA and sedentarism on cognition and brain activity. We hope this work will be considered a step forward in searching ways to improve children and adolescents’ health and to reduce educational and overall inequalities in developing countries.

## Additional file

Additional file 1 SPIRIT Checklist. (DOC 123 kb)

## Data Availability

Not applicable.

## Abbreviations

ACC: Accelerometry
BMR: Basal metabolic rate
C-HIIT: Cooperative High-Intensity Interval Training
DRI: Diffusion Tensor Imaging
EEG: Electroencephalography
fMRI: Functional magnetic resonance imaging
GPA: Grade point average
HEI: Healthy Eating Index
HRV: Heart rate variability
IFIS: International Fitness Scale
INTA: Instituto de Nutrición y Tecnología de los Alimentos
KIDMED: Mediterranean Diet Quality Index
METs: Metabolic equivalents
MICT: Moderate-Intensity Continuous Training
MRI: Magnetic resonance imaging
MVPA: Moderate-to-vigorous-intensity physical activity
NCPT: NeuroCognitive Performance Test
PA: Physical activity
PACO: Pedalea y Anda al Colegio
PE: Physical education
PHV: Peak height velocity
PROFITH: PROmoting FITness and Health through physical activity
RCT: Randomized controlled trial
SC: Sedentary condition
SIMCE: System for Assessment of Educational Quality
SPAQ: School context physical activity
SSR: Sleep Self-Report
SVI: School vulnerability index
YAP-SL: Youth Activity Profile-Spain Latin America
