# Correction to: Study protocol and rationale of the “Cogniaction project” a cross-sectional and randomized controlled trial about physical activity, brain health, cognition, and educational achievement in schoolchildren

**DOI:** 10.1186/s12887-021-02718-9

**Published:** 2021-05-28

**Authors:** Patricio Solis-Urra, Jorge Olivares-Arancibia, Ernesto Suarez-Cadenas, Javier Sanchez-Martinez, Fernando Rodríguez-Rodríguez, Francisco B. Ortega, Irene Esteban-Cornejo, Cristina Cadenas-Sanchez, Jose Castro-Piñero, Alejandro Veloz, Steren Chabert, Kabir P. Sadarangani, Juan Pablo Zavala-Crichton, Jairo H. Migueles, Jose Mora-Gonzalez, Milton Quiroz-Escobar, Diego Almonte-Espinoza, Alfonso Urzúa, Constantino D. Dragicevic, Aland Astudillo, Eduardo Méndez-Gassibe, Daniel Riquelme-Uribe, Marcela Jarpa Azagra, Carlos Cristi-Montero

**Affiliations:** 1 IRyS Research Group, School of Physical Education, Pontificia Universidad Católica de Valparaiso, Viña del Mar, Chile; 2 Department of Physical and Sports Education, Faculty of Sport Sciences, PROFITH “PROmoting FITness and Health through physical activity” Research Group, Sport and Health University Research Institute (iMUDS), University of Granada, Granada, Spain; 3 Department of Physical Education, Faculty of Sciences of Sport and Physical Education, University of A Coruña, A Coruña, Spain; 4 Physical Education School, Universidad de Las Américas, Viña del Mar, Chile; 5 Faculty of Sport Science, Pablo de Olavide University, Seville, Spain; 6 Departament of Physical Education, Faculty of Education Sciences, University of Cádiz, Puerto real, Spain; 7 Biomedical Engineering Department, Universidad de Valparaíso, Valparaíso, Chile; 8 CINGS, Centro de Investigación en Ingeniería para la Salud, Universidad de Valparaíso, Valparaíso, Chile; 9 School of Kinesiology, Faculty of Health Sciences, Universidad San Sebastián, Santiago, Chile; 10 Escuela de Kinesiología, Facultad de Salud y Odontología, Universidad Diego Portales, Santiago, Chile; 11 Facultad de Educación y Ciencias Sociales, Universidad Andrés Bello, Viña del Mar, Chile; 12 Independent Imagenology Center Quintaimagen, Viña del Mar, Chile; 13 School of Psychology, Universidad Católica del Norte, Antofagasta, Chile; 14 Auditory and Cognition Center, Universidad de Chile, Santiago, Chile; 15 Sports and Exercise Medicine Resident, Universidad Mayor, Santiago, Chile; 16 Universidad Adventista de Chile, Chillan, Chile; 17 Center for Research, Development and Innovation APLICAE, Santiago, Chile; 18 School of Pedagogy, Pontificia Universidad Católica de Valparaiso, Valparaíso, Chile

**Correction to: BMC Pediatr 19, 260 (2019)**

**https://doi.org/10.1186/s12887-019-1639-8**

Following the publication of the original article [1], the authors noticed that one of the author names has been incorrectly captured. Kabir P. Saradangani should be Kabir P. Sadarangani. Correct name is shown in the author group section above.

The original article has been corrected.
